# Embryonic Expression of Nras^G 12 D^ Leads to Embryonic Lethality and Cardiac Defects

**DOI:** 10.3389/fcell.2021.633661

**Published:** 2021-02-11

**Authors:** Xiaona You, Myung-Jeom Ryu, Eunjin Cho, Yanzhi Sang, Alisa Damnernsawad, Yun Zhou, Yangang Liu, Jing Zhang, Youngsook Lee

**Affiliations:** ^1^McArdle Laboratory for Cancer Research, University of Wisconsin-Madison, Madison, WI, United States; ^2^Department of Cell and Regenerative Biology, University of Wisconsin-Madison, Madison, WI, United States

**Keywords:** *Nras* mutation, RASopathy, congenital heart defects, fetal liver hematopoiesis, embryonic development, Noonan syndrome, heart development

## Abstract

Ras proteins control a complex intracellular signaling network. Gain-of-function mutations in *RAS* genes lead to RASopathy disorders in humans, including Noonan syndrome (NS). NS is the second most common syndromic cause of congenital heart disease. Although conditional expression of the *Nras^G12D/^*^+^ mutation in adult hematopoietic system is leukemogenic, its effects on embryonic development remain unclear. Here, we report that pan-embryonic expression of endogenous *Nras^G12D/^*^+^ by Mox2-Cre in mice caused embryonic lethality from embryonic day (E) 15.5 and developmental defects predominantly in the heart. At E13.5, *Nras^G12D/^*^+^*; Mox2^Cre/^*^+^ embryos displayed a moderate expansion of hematopoietic stem and progenitor cells without a significant impact on erythroid differentiation in the fetal liver. Importantly, the mutant embryos exhibited cardiac malformations resembling human congenital cardiac defects seen in NS patients, including ventricular septal defects, double outlet right ventricle, the hypertrabeculation/thin myocardium, and pulmonary valve stenosis. The mutant heart showed dysregulation of ERK, BMP, and Wnt pathways, crucial signaling pathways for cardiac development. Endothelial/endocardial-specific expression of *Nras^G12D/^*^+^ caused the cardiac morphological defects and embryonic lethality as observed in *Nras^G12D/^*^+^*; Mox2^Cre/^*^+^ mutants, but myocardial-specific expression of *Nras^G12D/^*^+^ did not. Thus, oncogenic *Nras^G12D^* mutation may not be compatible with embryonic survival.

## Introduction

Normal cardiac development is critical for proper cardiac function and embryo viability, which is regulated by complex biological processes including signaling transduction pathways. Congenital cardiac defects are one of the most common forms of human birth defects (Benjamin et al., 2017). However, the genetic basis responsible for congenital cardiac defects is not fully understood. The identification of molecular pathways critical for normal heart development could potentially lead to mechanistic discoveries underlying human congenital heart disease as well as novel therapies. Noonan syndrome (NS) is an autosomal dominant disorder with an incident of 1:1,000-2,500 live births (Baban et al., 2019). It is characterized by multiple defects with variable penetrance, including cranial facial abnormalities and congenital heart defects. The most frequent cardiac manifestation is pulmonic stenosis resulting from dysplastic valve leaflets, but atrioventricular or ventricular septal defects, mitral valve abnormalities and hypertrophic cardiomyopathy are also observed (Lauriol et al., 2015). NS patients have significantly higher risk to develop juvenile myelomonocytic leukemia (JMML). NS, along with a few other developmental diseases, belongs to RASopathy disorders, which are caused by genetic mutations hyperactivating the RAS/RAF/MEK/ERK pathway (Rauen, 2013). The mutated genes include all three *RAS* isoforms (*HRAS, NRAS*, and *KRAS*), RAS function modulators (*PTPN11, SOS1, SHOC2, NF1*, and *SPRED1*), and downstream signaling transducers (*RAF1, BRAF, MEK1*, and *MEK2*) (Cirstea et al., 2010).

Ras proteins are highly conserved small GTPases and act as master signaling switches that couple extracellular stimuli to the intracellular response machinery. They cycle between active GTP-bound state and inactive GDP-bound state (Bourne et al., 1990). Activated Ras proteins subsequently activate multiple downstream signaling pathways, including the RAF/MEK/ERK pathway. Canonical oncogenic mutations at RAS G12, G13, and Q61 severely compromise the hydrolysis of GTP to GDP, leading to the accumulation of Ras-GTP and the hyperactivation of Ras signaling. These strong mutations are identified essentially in all human cancers (Bos, 1989). Notably, more than 80% of patients with Costello syndrome (CS), a RASopathy disorder, harbor a heterozygous germline G12 mutation in *HRAS* (Aoki et al., 2005; Gripp et al., 2006; Kerr et al., 2006). A mouse CS model with endogenous expression of *Hras^G12V/^*^+^ induces developmental defects and neoplasms as seen in CS patients (Chen et al., 2009). In addition to CS-like phenotypes, mice with the *Hras^G12S/^*^+^ mutation are resistant to high-fat diet-induced obesity and exhibit impaired hepatic energy homeostasis (Oba et al., 2018).

In contrast to *HRAS*, relatively weak mutations in the *KRAS* gene are identified in RASopathy disorders (e.g., NS) (Schubbert et al., 2006; Cirstea et al., 2010). Five weak *KRAS* mutations (V14I, P34R, T58I, D153V, and F156L) associated with NS were previously characterized (Schubbert et al., 2007). P34R and D153V have normal intrinsic GTPase activity, while V14I and T58I have less impaired intrinsic GTPase activity compared to oncogenic G12D. Although F156L has similarly impaired intrinsic GTPase activity as G12D, this defect is partially rescued by its increased nucleotide exchange activity. These observations suggest that strong oncogenic *KRAS* mutations are not tolerated during human development. Indeed, expression of *Kras^G12D/^*^+^ in the mouse germline is embryonic lethal, with mutants dying at midgestation stages (Tuveson et al., 2004). To date, numerous unrelated individuals and NS families have been identified with *NRAS* mutations, including T50I, G60E, and a few G12 mutations (Cirstea et al., 2010; Denayer et al., 2012; Kraoua et al., 2012; Altmuller et al., 2017; Garren et al., 2020). Similar to *KRAS* mutations, NS-associated *NRAS* T50I and G60E mutations are weaker mutations compared to G12V as they only marginally activate MEK/ERK signaling in serum-deprived cells (Cirstea et al., 2010; Runtuwene et al., 2011; Denayer et al., 2012; Kraoua et al., 2012; Ekvall et al., 2015). By contrast, the NS patients with *NRAS* G12 mutations (G12N, G12R, G12S, and G12D) display more severe phenotypes than typical NS, including JMML, brain tumors, or neonatal lethality (Altmuller et al., 2017; Garren et al., 2020).

We and others previously showed that mice with the knock-in *Nras^G12D^* mutation are leukemogenic (Wang et al., 2010a, 2011b; Li et al., 2011; Xu et al., 2013). The conditional expression of endogenous Nras^G12D^ in adult hematopoietic system by Mx1-Cre (Wang et al., 2010a, 2011b; Li et al., 2011; Xu et al., 2013) induces a fully penetrant hematologic malignancy after a prolonged latency. However, when early pan-embryonic expression of *Nras^G12D/^*^+^ was induced using Mox2-Cre that expresses Cre in epiblasts beginning at E5, we could not obtain any live pups, suggesting that early ubiquitous embryonic expression of Nras^G12D^ leads to embryonic lethality (Wang et al., 2011b). However, the roles of Nras^G12D^ remain unknown during embryonic development. Here, we demonstrate that pan-embryonic expression of *Nras^G12D/^*^+^ driven by Mox2-Cre results in embryonic lethality between E15.5 and E17.5. At E13.5, mutant embryos appear grossly normal. However, further analyses indicate a moderate expansion of hematopoietic stem and progenitor cells in the fetal liver. Importantly, prominent heart morphological defects are detected from E13.5, which resemble human congenital heart defects such as NS. E13.5 mutant hearts showed downregulated non-canonical Wnt and BMP pathways, and hyper-activated MEK/ERK signaling in the heart. Using different Cre lines to drive Nras^G12D^ expression in different cardiac cell types, we discovered that the cardiac defects and embryonic lethality observed in *Nras^G12D/^*^+^*; Mox2^Cre/^*^+^ mutants originated from endothelial/endocardial cells, but not from myocardial cells.

## Materials and Methods

### Generation of Mutant Mice

All mouse lines were maintained on a pure C57BL/6 genetic background, which was achieved by more than six backcrosses to a C57BL/6 background. Mice bearing the conditional oncogenic *Nras* mutation (*Nras^loxp stop cassett loxp (LSL) G12D/^*^+^) (Haigis et al., 2008) were crossed to *Mox2*^Cre/+^ mice (Tallquist and Soriano, 2000) (Jackson Laboratory #003755) to generate *Nras^LSL G12D/^*^+^*; Mox2^Cre/^*^+^ (*Nras^G12D/^*^+^*; Mox2^Cre/^*^+^) embryos. *Tie2-Cre* or *cTnt* (*Tnnt2)-Cre* mice (Jackson Laboratory Stock #008863 or 024240) were also employed to generate endothelial/endocardial- or myocardial-specific *Nras^G12D/^*^+^ mutants, respectively. Embryos were genotyped for the *Nras* mutation and *Cre* alleles as described (Wang et al., 2011b). All animal experiments were conducted in accordance with the *Guide for the Care and Use of Laboratory Animals* and approved by an Animal Care and Use Committee at the University of Wisconsin (protocol #M005328). The program is accredited by the Association for Assessment and Accreditation of Laboratory Animal Care. Additional methods are described in Supplementary Materials and Methods.

## Results

### Pan-Embryonic Expression of *Nras^G12D/^*^+^ Leads to Embryonic Lethality Between E15.5 and E17.5

**FIGURE 1:**
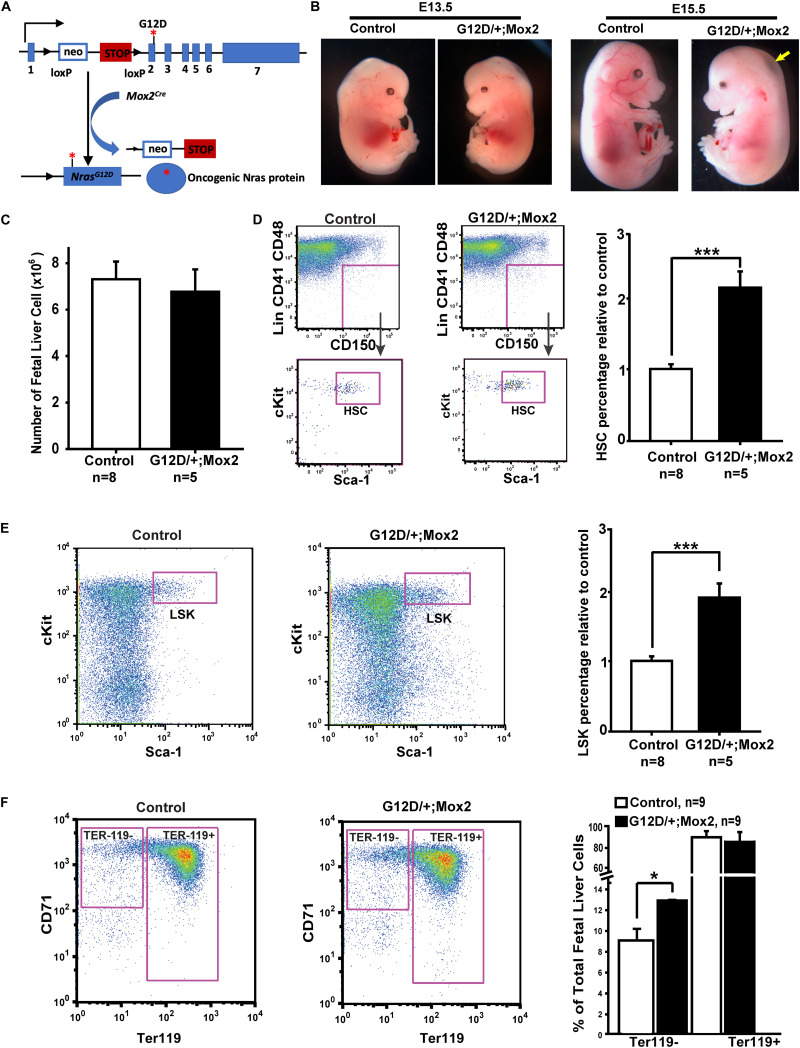
*Nras^G12D/^*^+^*; Mox2^Cre/^*^+^ (G12D/ +; Mox2) embryos exhibit whole body edema and the small liver at E15.5. **(A)** Schematic illustration of floxed and activated oncogenic *Nras* alleles. **(B)** Representative images of E13.5 and E15.5 WT control and G12D/ +; Mox2 mutant. The G12D/ +; Mox2 embryo looks grossly normal at E13.5 but at E15.5 looks pale and shows whole body edema as indicated by an arrow. **(C)** Quantitative analysis of cell numbers in E13.5 fetal livers from G12D/ +; Mox2 vs. control embryos. **(D)** Flow cytometric analysis and quantification of HSCs (defined as Lin^–^ CD41^–^ CD48^–^ Mac1^+^ CD150^+^ Sca1^+^ cKit^+^ cells) in total live fetal liver cells. HSC percentages in control fetal liver cells were arbitrarily set as 1. **(E)** Flow cytometric analysis and quantification of LSKs (defined as Lin^–^ Sca1^+^ cKit^+^ cells) in total fetal liver live cells. LSK percentage in control fetal liver cells were arbitrarily set as 1. **(F)** Flow cytometric analysis and quantification of erythroid differentiation based on CD71 and Ter119 expression. Unpaired 2-tailed Student’s t tests were used to determine the significance. Results are presented as mean + SD. *p < 0.05; ***p < 0.001.

We used a conditional oncogenic *Nras* mouse line (*Nras^Loxp–STOP–Loxp (LSL)^^G12D/^*^+^ line) to induce oncogenic Nras expression from its endogenous locus in somatic cells (Haigis et al., 2008). In this mouse line, the expression of oncogenic Nras is silenced by a floxed stop cassette upstream of the coding sequence in the absence of Cre and induced upon temporally and spatially controlled Cre expression (Figure 1A). We previously reported that pan-embryonic expression of *Nras^G12D/^*^+^ driven by *Mox2*^Cre^ beginning at E5 resulted in no live neonatal mice (Wang et al., 2011b), suggesting embryonic lethality of the mutants. To determine the exact stage of embryonic lethality, we harvested embryos between E12.5 and E17.5 from crosses of *Nras^LSL G12D/^*^+^ and *Mox2*^Cre/+^ mice (Table 1A and see section “Materials and Methods”). Expected numbers of *Nras^G12D/^*^+^*; Mox2^Cre/^*^+^ were recovered between E12.5 and E14.5. However, the total number of mutant embryos as well as the number of live mutants were significantly reduced from E15.5. All *Nras^G12D/^*^+^*; Mox2^Cre/^*^+^ embryos were dead by E17.5. Therefore, our data indicate that pan-embryonic expression of *Nras^G12D/^*^+^ results in embryonic lethality between E15.5 and E17.5.

**TABLE 1A:** Embryonic lethality of *Nras^G12D/^*^+^*^;^ Mox2-Cre* mutants.

**Embryo stage**	**No of total live embryos**	**Expected No of live mutant embryos**	**Recovered No of live mutant embryos (total mutant embryos)**	***p*-value**
E12.5	29	7	4(4)	*p* = 0.31
E13.5	100	25	19(19)	*p* = 0.31
E14.5	22	5–6	5(6)^#^	*p* = 0.73
E15.5	46	11–12	4(6)^#^	*p* = 0.028
E16.5	51	13	3(7)^#^	*p* = 0.0073
E17.5	61	15	0(3)^#^	*p* = 0.0001

*^#^Some dead embryos were genotyped as the mutant, while others could not be genotyped due to resorption/necrosis.*

To determine the mechanisms contributing to embryonic lethality in *Nras^G12D/^*^+^*; Mox2^Cre/^*^+^embryos, we carefully examined the histology of the embryos. The mutant embryos were grossly normal at E13.5 as compared to the wild-type (WT) littermate control (Figure 1B). However, the mutant embryos at E15.5 were smaller and pale with whole-body edema (arrow in Figure 1B) as compared to the control. Evaluation of multiple tissues, including the lung, kidney, and brain did not reveal any gross morphological defects (our unpublished observations). However, the fetal livers from mutant embryos were smaller at E15.5 and hepatic necrosis was evident as indicated by arrow, especially in the regions distal from blood flow (Supplementary Figure 1). Therefore, we investigated whether the embryonic lethality is caused by defective fetal liver hematopoiesis.

### *Nras^G12D/^*^+^*; Mox2^Cre/^*^+^ Embryos Display an Expansion of Hematopoietic Stem and Progenitor Cells in the E13.5 Fetal Liver

We examined fetal liver hematopoiesis in E13.5 *Nras^G12D/^*^+^*; Mox2^Cre/^*^+^ and control embryos. At this stage, the number of fetal liver cells in mutant embryos was indistinguishable from WT control littermates (Figure 1C). However, detailed quantification of fetal liver hematopoietic stem cells (HSCs, defined as Lin^–^ Mac1^+^ CD41^–^ CD48^–^ CD150^+^ Sca1^+^ cKit^+^) and LSK cells (hematopoietic stem and progenitor cells, defined as Lin^–^ Sca1^+^ cKit^+^) indicated that both populations were expanded in mutant embryos (Figures 1D,E). This result is consistent with our prior analysis of hematopoietic stem and progenitor cells in adult *Nras^G12D/^*^+^*; Mx1-Cre* mice (Wang et al., 2010b, 2013a). We also examined fetal liver erythroid differentiation based on CD71 and Ter119 expression patterns as previously described (Zhang et al., 2003). Our results showed a moderate expansion of Ter119^–^ CD71^mid/hi^ cells, which were enriched with colony forming unit-erythroid (CFU-E) progenitors and early erythroblasts, while the percentage of terminally differentiating Ter119^+^ erythroid cells appeared comparable to that of control embryos (Figure 1F). This result is in sharp contrast to the blocked erythroid differentiation previously reported in *Kras^G12D/^*^+^ embryos (Zhang et al., 2007; Zhang and Lodish, 2007). Our data indicate that endogenous Nras^G12D/+^ signaling caused moderate effects on fetal liver hematopoiesis at E13.5. We previously reported that *Nras^Q61R/^*^+^*; Vav-Cre* mice, in which Nras^Q61R/+^ signaling is activated by Vav-Cre in the fetal liver from E11.5 (Ogilvy et al., 1999; Damnernsawad et al., 2016), were born at the expected Mendelian ratio (You et al., 2018). Since *Nras^Q61R^* is a much stronger mutant than *Nras^G12D^* (Kong et al., 2016), we believe that the mild perturbation of fetal liver hematopoiesis in *Nras^G12D/^*^+^*; Mox2^Cre/^*^+^ embryos is unlikely to cause a lethal phenotype.

### *Nras^G12D/^*^+^*; Mox2^Cre/^*^+^ Embryos Exhibit Cardiac Developmental Defects

We systematically examined serial sections of the lung, kidney, and brain, and did not find any significant morphological abnormalities (data not shown). Given that normal cardiac morphogenesis is critical for proper cardiac function and embryonic survival, and that hyperactivation of Ras signaling is associated with cardiac abnormalities in humans such as NS, we set out to determine whether cardiac development was defective in mutant embryos.

**FIGURE 2:**
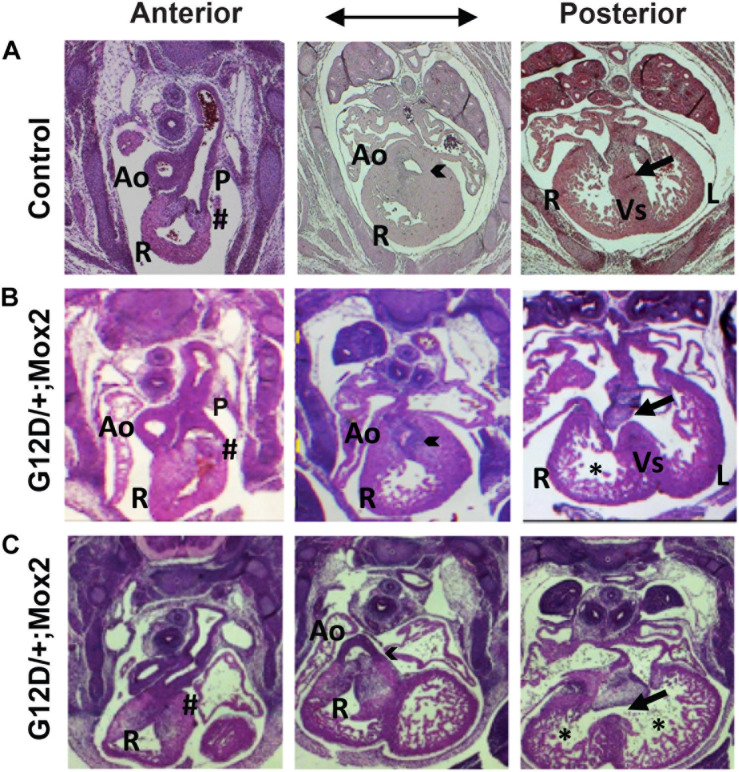
*Nras^G12D/^*^+^*; Mox2^Cre/^*^+^ (G12D/ +; Mox2) embryos exhibit cardiac developmental defects. Representative images of H&E stained transverse sections of control **(A)** vs. two G12D/ +; Mox2 mutant **(B,C)** embryos at E13.5. The mutant hearts show VSD (indicated by arrow in right panels **B,C**), DORV (indicated by arrowhead in middle panels **B,C**) and the thin ventricular wall (indicated by * in right panels **B,C**). The pulmonary valves were not remodeled properly (indicated by # in left panels **B,C**). Ao, Aorta; P, Pulmonary valve; R, Right ventricle; L, Left ventricle; Vs, Ventricular septum.

Cardiac development of *Nras^G12D/^*^+^*; Mox2^Cre/^*^+^embryos was analyzed by examining serial H&E stained transverse sections (Figure 2). Since the mutants exhibited embryonic lethality between E15.5 and E17.5, we examined the mutant hearts at E13.5 for cardiac morphological defects. At E13.5, the interventricular septum in the control heart fused to the endocardial cushion and thus the right ventricle was separated from the left ventricle (right panel in Figure 2A, indicated by arrow). Conversely, the interventricular septum in the mutant heart failed to fuse with the endocardial cushion, leading to ventricular septal defects (VSDs) (right panels in Figures 2B,C, indicated by arrows), which is a common human congenital cardiac defect. In the normal heart, the aorta exits the left ventricle and the pulmonary trunk exits the right ventricle as shown in the left and middle panels of Figure 2A. However, the mutant heart showed a double outlet right ventricle (DORV) where both the aorta and pulmonary trunk exited the right ventricle (arrowheads, middle panels in Figures 2B,C), which is one of the human congenital cardiac defects. The mutant hearts showed an aorta that was connected to the right ventricle (middle panels in Figures 2B,C) in contrast to the control, which showed no connection between aorta and right ventricle (middle panel in Figure 2A). In addition, the mutant heart exhibited thinning of the ventricular wall, where the thin compact layer and hypertrabeculation were observed compared to controls as indicated by asterisk (right panels in Figures 2B,C vs. right panel in Figure 2A). The compact layer thickness in the right and left ventricle in the mutant hearts was significantly reduced by 62.5 and 45.5%, respectively (Supplementary Figure 2D). Furthermore, pulmonary valve stenosis was observed in the mutant heart as compared to the control (as indicated by pound sign in left panels in Figures 2B,C vs. left panel in Figure 2A), indicating defects in pulmonary valve remodeling. VSDs were fully penetrant (6/6 mutant hearts), while DORV, thin myocardium, or pulmonary stenosis was partially penetrant (4/6 mutant hearts).

Ras signaling pathways are known to regulate cell proliferation and survival, which are crucial for normal cardiac development. Thus, cell proliferation and apoptosis were investigated in the embryonic heart. To examine cell proliferation, immunohistochemistry was performed on heart sections at E13.5 using Ki-67 antibodies (Supplementary Figure 2A). Our results showed no significant alterations in cell proliferation in the mutant heart compared to the control heart. When the trabecular and compact layers of the right and left ventricles were analyzed separately, we did not detect any significant changes in cell proliferation between the mutant heart and the control heart (Supplementary Figures 2B,C). Furthermore, the mutant heart did not show any significant changes in apoptosis compared to the control at E13.5 by immunofluorescence staining using Caspase3 antibodies (Supplementary Figure 3).

### *Nras^G12D/^*^+^*; Mox2^Cre/^*^+^ Hearts Exhibit Defective Developmental Signaling Pathways and the Hyperactivated MEK/ERK Pathway

**FIGURE 3:**
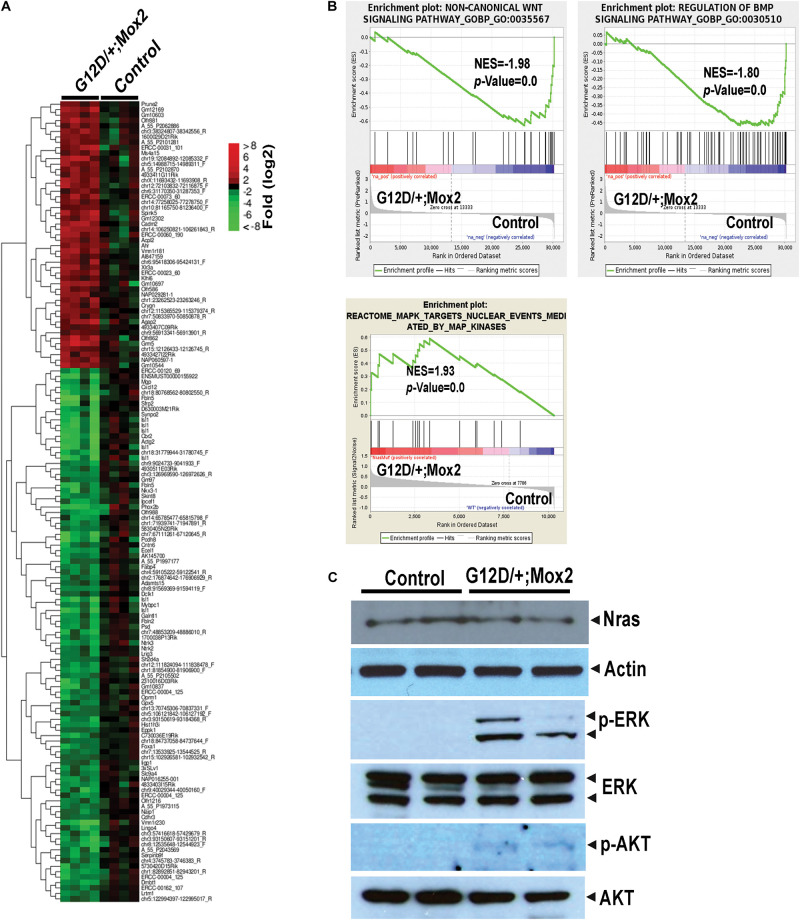
Global gene expression analyses of E13.5 *Nras^G12D/^*^+^*; Mox2^Cre/^*^+^ (G12D/^+^; Mox2) heart. Agilent microarray analysis was performed on E13.5 hearts from control and G12D/ +; Mox2 embryos. **(A)** Heat map depicting differentially expressed genes (*p* < 0.01 and fold change >2) between control and mutant hearts. **(B)** Gene Set Enrichment Analysis identified altered gene expression profiles in mutant hearts. Representative examples in non-canonical Wnt signaling pathway, BMP signaling pathway, and MAPK cascade are shown here. NES, normalized enrichment score. **(C)** Analysis of MEK/ERK and AKT signaling in E13.5 mutant hearts by Western blotting. Actin antibody was used for a loading control.

Our analyses of embryonic organ development indicate that the most significant defects were observed in the developing heart. To understand the molecular mechanisms underlying cardiac defects in *Nras^G12D/^*^+^*; Mox2^Cre/^*^+^ embryos, we performed gene profiling analysis on E13.5 control and mutant hearts. We identified 147 genes that were differentially expressed in mutant hearts (*p* < 0.01 and fold change >2), 98 genes downregulated and 49 genes upregulated (Figure 3A). One of the downregulated genes in mutant hearts is *Islet1* (*Isl1*) (24-fold reduction), which is known to play critical roles in cardiac development (Moretti et al., 2006) and is also involved in conduction system development (Liang et al., 2015). Gene set enrichment analysis (GSEA) showed that non-canonical Wnt and BMP signaling pathways were significantly downregulated in mutant hearts (Figure 3B). Both Wnt (canonical and non-canonical) and BMP signaling pathways play critical roles in early embryonic as well as cardiac development (Tian et al., 2010; Wang et al., 2011a). By contrast, the MEK/ERK pathway downstream of oncogenic Ras was hyperactivated as expected due to the expression of *Nras^G12D/^*^+^. The hyperactivation of MEK/ERK signaling in mutant hearts was further validated using Western blot analysis (Figure 3C). Our finding is supported by previous reports that hyperactivated RAS/RAF/MEK/ERK pathway affects cardiac development in mice and humans [reviewed in Wang (2007)]. Phosphorylation of AKT (p-AKT), another signaling molecule downstream of Ras, was also moderately increased in the mutant heart, indicating that both ERK and AKT axes are activated.

### Endothelial/Endocardial Origin of Cardiac Defects in *Nras^G12D/^*^+^*; Mox2^Cre/^*^+^ Embryos

It is believed that crosstalk between the endocardium and myocardium is essential for normal heart development (MacGrogan et al., 2018) and the endocardium/endothelium plays an important role in underlying myocardial development (Mysliwiec et al., 2011). Thus, we investigated whether the cardiac defects of the mutant heart were due to endothelial/endocardial- or myocardial-specific defects by employing Tie2-Cre (Kisanuki et al., 2001) or Tnnt2-Cre (Chen et al., 2004) mice, respectively (Tables 1B,C and Supplementary Figure 4). *Nras^LSL G12D/^*^+^*; Tie2-Cre* (*Nras^G12D/^*^+^*; Tie2-Cre*) mutants showed the same embryonic lethality as *Nras^G12D/^*^+^*; Mox2^Cre/^*^+^ embryos (Table 1B). All *Nras^G12D/^*^+^*; Tie2-Cre* mutant embryos died by E17.5. Histological analyses at E13.5 revealed that the *Nras^G12D/^*^+^*; Tie2-Cre* mutant heart showed VSDs (2/5 mutants; indicated by arrow) and the thin compact layer with the deeply invaginated trabecular layer (3/5 mutants; indicated by asterisk) compared to the control heart (Supplementary Figure 4). DORV or pulmonary stenosis was also detected (2/5 mutants). Interestingly, *Nras^LSL^^G12D/^*^+^*; Tnnt2-Cre* (*Nras^G12D/^*^+^*; Tnnt2-Cre*) mutants showed neither embryonic lethality (Table 1C) nor any gross cardiac malformations (data not shown), indicating that the myocardial-specific expression of Nras^G12D^ does not contribute to cardiac malformations or lethality during embryonic development. These results indicate that cardiac defects observed in *Nras^G12D/^*^+^*; Mox2^Cre/^*^+^ embryos have originated from endothelial/endocardial defects, but not from myocardial defects.

**TABLE 1B:** Generation of *Nras ^G12D/^*^+^;*Tie2 ^Cre/^*^+^ mutants.

**Embryo stage**	**No of total live embryos**	**Expected No of live mutant embryos**	**Recovered No of live mutant embryos (total mutant embryos)**	***p*-value**
E14.5	14	3–4	4(4)	*p* = 0.66
E17.5	48	12	0(4)^#^	*p* = 0.0002

*^#^Some dead embryos were genotyped as the mutant, while others could not be genotyped due to resorption/necrosis.*

**TABLE 1C:** Generation of *Nras ^G12D/^*^+^;*Tnnt2 ^Cre/^*^+^ mutants.

**Genotype**	**No of pups/total**	**Mendelian ratio**	***p*-value**
WT	4/18	25%	*p* = 0.7
*Tnnt2 ^Cre/^*^+^	5/18	25%	*p* = 0.7
*Nras ^G12D/^*^+^	4/18	25%	*p* = 0.7
*Nras ^G12D/^*^+^; *Tnnt2 ^Cre/^*^+^	5/18	25%	*p* = 0.7

## Discussion

Here we report that pan-embryonic expression of *Nras^G12D/^*^+^ in mice caused embryonic lethality at E15.5-E17.5 and developmental defects predominantly in the heart. The E13.5 *Nras^G12D/^*^+^*; Mox2^Cre/^*^+^ mutants displayed a moderate expansion of hematopoietic stem and progenitor cells without significant impact on erythroid differentiation in the fetal liver. The development of other tissues, including the lung, kidney, and brain, appeared morphologically normal. Importantly, the mutant hearts showed cardiac malformations resembling human congenital cardiac defects seen in NS, such as VSDs, DORV, pulmonary valve stenosis, and hypertrabeculation/thin myocardium. Consistent with cardiac functional insufficiency, mutant embryos started to die at E15.5 with whole body edema and pale body. Thus, our data indicate that ubiquitous expression of *Nras^G12D/^*^+^ in the early mouse embryo results in a spectrum of cardiac malformations in the mid to late gestation period. These cardiac defects have been well accepted as lethal congenital heart defects in various mouse models (Shou et al., 1998; Lee et al., 2000; He et al., 2012).

Somatic mutations in *NRAS* are involved in the development of hematological malignancies and a variety of solid tumors (COSMIC database)^1^. However, these oncogenic mutations have rarely been identified as germline mutations in human live births, and the majority of identified *NRAS* mutations in NS patients are biochemically weaker mutations instead (Runtuwene et al., 2011). These data suggest that like *KRAS* mutations, strong oncogenic *NRAS* mutations would cause embryonic lethality in humans. In support of this notion, we showed that pan-embryonic expression of *Nras*^G12D/+^ at its endogenous level caused embryonic lethality in mice (Table 1), while the expression of a hypomorphic allele of Nras^G12D^ at up to 80% of Nras^G12D^ endogenous level did not cause any detectable phenotypes (Wang et al., 2011c).

*Nras^G12D/^*^+^*; Mox2^Cre/^*^+^ embryos at E13.5 were grossly normal (Figure 1B) and showed mild phenotypes in fetal liver hematopoiesis (Figure 1). We detected that hematopoietic stem and progenitor cells underwent moderate expansion without significantly affecting terminal erythroid differentiation. We previously reported that *Nras^Q61R/^*^+^*; Vav-Cre* mice, in which the hematopoietic-specific expression of Nras^Q61R^ [a much stronger mutant than Nras^G12D^ (Kong et al., 2016)] was induced in the fetal liver from E11.5 (Ogilvy et al., 1999; Damnernsawad et al., 2016), were born at normal Mendelian ratios with hematopoietic phenotypes (You et al., 2018) similar to *Nras^G12D/^*^+^*; Mox2^Cre/^*^+^ embryos or *Nras^G12D/+^; Mx1-Cre* adult mice (Wang et al., 2011b). Therefore, we believe that the hematopoietic phenotypes would not cause or contribute to the embryonic lethality observed in *Nras^G12D/^*^+^*; Mox2^Cre/^*^+^ mutants. It is likely that the smaller liver and liver necrosis observed in E15.5 *Nras^G12D/^*^+^*; Mox2^Cre/^*^+^ embryos are a secondary effect caused by cardiac defects, due to limited blood flow to support the survival of fetal liver cells.

Our data indicate that the endothelial/endocardial expression of *Nras^G12D/^*^+^ in *Nras^G12D/^*^+^*; Tie2-Cre* embryos caused embryonic lethality (Table 1B) and cardiac malformations (Supplementary Figure 4), recapitulating the defects observed in *Nras^G12D/^*^+^*; Mox2^Cre/^*^+^ mutant embryos. By contrast, the myocardial-specific expression of *Nras^G12D/^*^+^ in *Nras^G12D/^*^+^*; Tnnt2-Cre* mutants showed neither embryonic lethality (Table 1C) nor gross cardiac malformations, indicating a non-cardiomyocyte origin of the cardiac defects observed in the *Nras^G12D/^*^+^*; Mox2^Cre/^*^+^ mutant mice. The ventricular myocardial wall consists of three layers: the endocardium (the inner layer), the myocardium (the middle layer) and the epicardium (the outer layer). During cardiac development, the one-layered myocardium must expand to the multilayered ventricular myocardial wall, consisting of the inner trabecular and outer compact layer (Wagner and Siddiqui, 2007). Myocardial trabeculation refers to the process by which the endocardium induces the underlying cardiomyocytes to proliferate, migrate to form finger-like projections in the heart. We and others demonstrated that endocardial signaling to the underlying myocardium plays crucial roles in the myocardial growth and development (Grego-Bessa et al., 2007; Mysliwiec et al., 2011). Our study indicates that Ras signaling in endothelial/endocardial cells is critical for normal cardiac morphogenesis including ventricular septation and the ventricular myocardial formation. These findings reiterate the importance of crosstalk between endocardial/endothelial cells and myocardial cells for normal ventricle development. Our conclusion is supported by other reports that the cell types responsible for the cardiac defects in a NS mouse model with the *Ptpn11 D61* mutation are of endothelial/endocardial, but not cardiomyocyte origin (Araki et al., 2004; Araki et al., 2009). Although we have not observed significant changes in cell proliferation and apoptosis in the mutant ventricular wall at E13.5, it is plausible that cell proliferation or apoptosis may have been altered transiently and regionally within the mutant heart. Since Tie2-Cre drives oncogenic Nras expression in all endothelial cells, including both endocardium and endothelium of the heart, it cannot be used to determine direct changes in the endocardium vs. indirect changes in the myocardium upon activating Ras signaling. Future experiments employing an endocardial-specific Cre driver (Wu et al., 2012) may help address this question.

Similar to *Nras^G12D^*, germline *Kras^G12D^* mutation in mice also caused embryonic lethality (Tuveson et al., 2004). The mutants died much earlier, between E9.5 and E11.5, with profound developmental arrest and widespread apoptosis, including cardiomegaly and abnormal brain development. Interestingly, although mice with endogenous cardiomyocyte-specific Kras^G12D^ expression showed hyperactivation of myocardial ERK and AKT signaling, they were born in expected Mendelian ratios and appeared healthy with normal function, size, and histology of the heart (Dalin et al., 2014). These phenotypes are highly consistent with our mutants containing the myocardial-specific expression of Nras^G12D^. In addition to *NRAS* and *KRAS*, embryonic expression of the most common and potent *Ptpn11* mutation (*Ptpn11^E76K/^*^+^) also results in embryonic lethality at E11.5 (Xu et al., 2011). In contrast to *NRAS* and *KRAS*, strong oncogenic *HRAS* mutations are identified in CS (Aoki et al., 2005). Corroborating with human results, mice with germline expression of *Hras^G12V/^*^+^ were born at the expected Mendelian frequency with CS-like phenotypes (Chen et al., 2009). *Hras^G12V/^*^+^ embryos at E19 do not display any cardiopulmonary anomalies. However, most of them (>80%) died within 14 days after birth. The cause of this neonatal lethality is unknown since there are no abnormalities in the heart and lungs and no evidence of bleeding.

Future studies will be aimed at determining how dysregulated gene expression and signaling pathways cause cardiac defects and embryonic lethality, which will advance mechanistic insights into congenital heart defects including NS associated heart defects. *Isl1* was significantly downregulated in mutant hearts (24-fold). Isl-1 plays critical roles in early cardiac development and is expressed in the second heart field that gives rise to the right ventricle, outflow tract and atria and thus considered as a cardiac progenitor marker (Moretti et al., 2006). Furthermore, a new *ISL1* mutation predisposes to congenital DORV (Wang et al., 2019). Thus, it would be interesting to investigate the effect of Isl1 downregulation on cardiac abnormalities in the mutant heart. Our GSEA identified that non-canonical Wnt and BMP signaling pathways were significantly downregulated in mutant hearts (Figure 3B). Since both Wnt and BMP signaling play critical roles in cardiac development (Tian et al., 2010; Wang et al., 2011a), future direction would be to determine their effects on gene regulation and thus cardiac malformations. Interestingly, our gene expression profiling showed the significant reduction of a Wnt regulator *Sfrp2* (Lin et al., 2016) and *Foxa1*(Wang et al., 2013b), a transcription factor targeted by BMP or Wnt (11.3- and 14.9-fold reduction, respectively). However, the mechanisms by which these alterations cause cardiac malformations remain to be elucidated. Wnt signaling plays key roles in early cardiac development and disease, but their functions are complex (Wagner and Siddiqui, 2007). Non-canonical Wnt such as Wnt11 induces cardiogenesis while other canonical Wnt3a and 8c inhibit this process during early development. BMPs are also involved in early embryonic patterning and cardiogenesis (Wagner and Siddiqui, 2007; Wang et al., 2011a). In particular, BMPs 2 and 4 are necessary for early cardiac mesoderm development, and BMP10 is crucial for ventricular trabeculation (Chen et al., 2004). Trabeculation is a critical step for the formation of normal thickened ventricular wall. It is plausible that reduced BMP signaling contributes to the thin ventricular wall in *Nras^G12D/^*^+^*; Mox2^Cre/^*^+^ heart. Furthermore, it would be interesting to investigate whether dysregulated Isl1, BMP, or Wnt signaling also plays a role in cardiac defects manifested in NS patients with RAS activation, which will help develop treatment options.

Taken together, our and others’ studies suggest that hyperactivation of Ras signaling beyond a certain threshold would cause cardiac defects and more severely, embryonic lethality. Furthermore, endothelial/endocardial-specific expression of the *Nras^G12D^* mutant gene results in embryonic lethality and contributes to the cardiac morphological defects observed in the *Nras^G12D/^*^+^*; Mox2^Cre/^*^+^ mutant hearts. Our findings would provide the important basis to advance our understanding of developmental defects associated with hyperactivated Ras signaling.

## Data Availability Statement

The original contributions presented in the study are included in the article/Supplementary Material, further inquiries can be directed to the corresponding author/s.

## Ethics Statement

All animal experiments were conducted in accordance with the Guide for the Care and Use of Laboratory Animals and approved by an Animal Care and Use Committee at the University of Wisconsin (protocol #M005328). The program is accredited by the Association for Assessment and Accreditation of Laboratory Animal Care.

## Author Contributions

XY, M-JR, JZ, and YLe: conception, design, writing, review, and/or revision of the manuscript. XY, M-JR, EC, YS, and AD: acquisition of data, analysis, and interpretation of data. YLi and YZ: technical or material support. JZ and YLe: study supervision. All authors contributed to the article and approved the submitted version.

## Conflict of Interest

The authors declare that the research was conducted in the absence of any commercial or financial relationships that could be construed as a potential conflict of interest.

## Footnotes

1 http://cancer.-sanger.ac.uk/
