# Supplementary material for: Embryonic Expression of NrasG 12 D Leads to Embryonic Lethality and Cardiac Defects
Source: Front Cell Dev Biol. 2021 Feb 11;9:633661. doi: 10.3389/fcell.2021.633661 (PMC7928391; doi:10.3389/fcell.2021.633661)
Supplement: Supplementary file 1 [file Data_Sheet_1.pdf]

Supplemental Information

Supplemental Materials and Methods

Preparation of mouse embryos, histopathology and immunofluorescence staining

Mutant embryos were obtained from timed-matings of *Nras*$^{LSL\ G12D/+}$ and different *Cre* mice. Pregnant females were euthanized with CO_2 and the uterus were removed into IMDM containing 10% FBS. Embryo viability was determined under microscope based on heart beating. Live embryos were photographed prior to fixation or dissection using digital camera (Nikon) attached to dissection microscopy (Zeiss). Tissues were harvested in cold PBS for protein and RNA extraction or IMDM containing 10% FBS for flow cytometry. For histopathology, embryos were fixed with 4% paraformaldehyde (PFA), paraffin embedded, cut at 5 μm with transverse sections, and stained with hematoxylin and eosin (H&E). Immunostaining was done as described (Cho et al., 2019). In brief, primary antibodies were used against cleaved Caspase-3 (1:200 dilution; Cell signaling) or Ki67 (1:500; DAKO) or MF20 (1:500; DSHB) followed by appropriate fluorescence or horse radish-conjugated secondary antibodies (Jackson Immuno Research). Slides were counterstained with DAPI (Vector) and mounted in Fluoromount G (Electron Microscopy Sciences). The sections were examined using epifluorescence microscopy (Zeiss Axiovert 200) connected to an AxioCam HRc camera.

Microarray analysis

Total RNAs were isolated from 4 pairs of E13.5 mutant and control heart tissues using RNeasy mini Kit (Qiagen). Microarray was performed using the whole mouse genome array (SurePrint G3 CGH/CGH+SNP 8x60K Bundle; Agilent Technologies). Statistical analysis of the obtained data was performed using Edge^3 software (Vollrath et al., 2009) to identify differentially expressed genes. Gene Set Enrichment Analysis (GSEA) was used to identify specific pathway changes in microarray data sets.

Western blot analysis and flow cytometry

Whole heart lysates were prepared from E13.5 heart tissues and Western blot was performed as previously described (Ryu et al., 2012). In brief, heart tissues were isolated from E13.5 embryos and lysed by sonication in lysis buffer containing 7M urea, 2M thiourea, 4% CHAPS, 130mM dithiothreitol, a complete protein inhibitor cocktail (Roche), and 1 mM NaF and Na_2VO_3. Protein lysates were resolved on 4-12% polyacrylamide gels (Invitrogen) in MOPS buffer. The primary antibodies against following antigens were used in this study: Nras (1:200 dilution; Santa Cruz), α-actin (1:5000; Sigma-Aldrich), and ERK, p-ERK, Akt, and p-Akt (1:1000; Cell Signaling Technologies). Flow cytometry was performed as described (Johnson et al., 2015). Briefly, the fetal liver cells from E13.5 embryos were resuspended in PBS with 2% FBS. Surface proteins were detected with fluorescein isothiocyanate (FITC)-conjugated antibodies B220 (RA3-6B2), CD3 (145-2C11), CD4 (GK1.5), CD5 (53-7.3), CD8 (53-6.7), CD41 (eBipMWReg30), CD48 (HM48-1), Gr-1 (RB6-8C5), and TER119 (TER-119); phycoerythrin (PE)-conjugated antibodies CD71 (R17217), CD150 (TC15-12F12.2) and Sca-1 (D7); PECy7-conjugated Mac1 (M1/70); PerCP-Cy5.5-conjugated Sca-1 (D7); and allophycocyanin (APC)-conjugated c-Kit (2B8) antibodies. Lineage markers were stained with FITC-conjugated B220, CD3, CD4, CD5, CD8, Gr-1 and TER119 antibodies. All antibodies were purchased from eBiosciences. The stained cells were

collected on a FACSCalibur or LSRII flow cytometer (BD Biosciences). The data were analyzed using FlowJo v9.0.2 software (TreeStar).

References

Cho, E., Kang, H., Kang, D.K., and Lee, Y. (2019). Myocardial-specific ablation of Jumonji and AT-rich interaction domain-containing 2 (Jarid2) leads to dilated cardiomyopathy in mice. *J Biol Chem* 294(13)**,** 4981-4996. doi: 10.1074/jbc.RA118.005634.

Johnson, K.D., Kong, G., Gao, X., Chang, Y.I., Hewitt, K.J., Sanalkumar, R., et al. (2015). Cis-regulatory mechanisms governing stem and progenitor cell transitions. *Sci Adv* 1(8)**,** e1500503. doi: 10.1126/sciadv.1500503.

Ryu, M.J., Liu, Y., Zhong, X., Du, J., Peterson, N., Kong, G., et al. (2012). Oncogenic Kras expression in postmitotic neurons leads to S100A8-S100A9 protein overexpression and gliosis. *J Biol Chem* 287(27)**,** 22948-22958. doi: 10.1074/jbc.M112.357772.

Vollrath, A.L., Smith, A.A., Craven, M., and Bradfield, C.A. (2009). EDGE(3): a web-based solution for management and analysis of Agilent two color microarray experiments. *BMC Bioinformatics* 10**,** 280. doi: 1471-2105-10-280 [pii]
10.1186/1471-2105-10-280.

Supplemental Figure Legends

Supplemental Figure 1. *Nras*$^{G12D/+}$*; Mox2*$^{Cre/+}$ (G12D/+; Mox2) embryos exhibit small liver and hepatic necrosis at E15.5. Representative H&E sections of the fetal livers from G12D/+; Mox2 and control littermates. Arrows indicate hepatic necrosis.

Supplemental Figure 2. *Nras*$^{G12D/+}$*; Mox2*$^{Cre/+}$ (G12D/+; Mox2) embryos do not show significant changes in cell proliferation in the heart. A. Cell proliferation assays by Ki-67 immunohistochemical staining on transverse heart sections from E13.5 embryos. The red line divides the inner trabecular and outer compact layer. RV, right ventricle; LV, left ventricle. B. To quantify proliferating cells, Ki-67 positive cells were counted per slide in different regions of the heart. RC, right ventricle compact layer; RT, right ventricle trabecular layer; LC, left ventricle compact layer, LT, left ventricle trabecular layer. C. A total number of Ki-67 positive cells per ventricle (Vent) were calculated by adding all the Ki-67 positive cells from each region. D. Thickness of the ventricular compact layer in mutants was quantitated as compared to controls at E13.5. The distance from the epicardium to a line between the trabecular and compact layer as shown in supplemental Fig S2A was measured in three sections per heart. n=3-4. Results are presented as mean +/- SD. * $p < 0.05$; ** $p < 0.01$.

Supplemental Figure 3. *Nras*$^{G12D/+}$*; Mox2*$^{Cre/+}$ (G12D/+; Mox2) embryonic hearts do not show significant changes in apoptosis. Activated caspase was detected by caspase 3 immunofluorescence staining on E13.5 heart sections. The sections were co-immunostained with

MF20 antibody, a cardiac muscle marker. Arrows indicate caspase 3 positive cells. Nuclear staining was done using Hoechst dye.

Supplemental Figure 4. Endothelial/endocardial origin of cardiac defects in *$Nras^{G12D/+}$; $Tie2^{Cre/+}$* mutants. Cardiac malformations in *$Nras^{G12D/+}$; Tie2-Cre* (G12D/+; Tie2) mutants shown by representative H&E stained transverse sections as compared to control at E14.5. The G12D/+; Tie2 embryo shows VSD (indicated by arrow) and the thin compact layer/hypertrabeculation (indicated by asterisk) compared to control.
