# Supplementary figures and images for: Embryonic Expression of NrasG 12 D Leads to Embryonic Lethality and Cardiac Defects
Source: Front Cell Dev Biol. 2021 Feb 11;9:633661. doi: 10.3389/fcell.2021.633661 (PMC7928391; doi:10.3389/fcell.2021.633661)

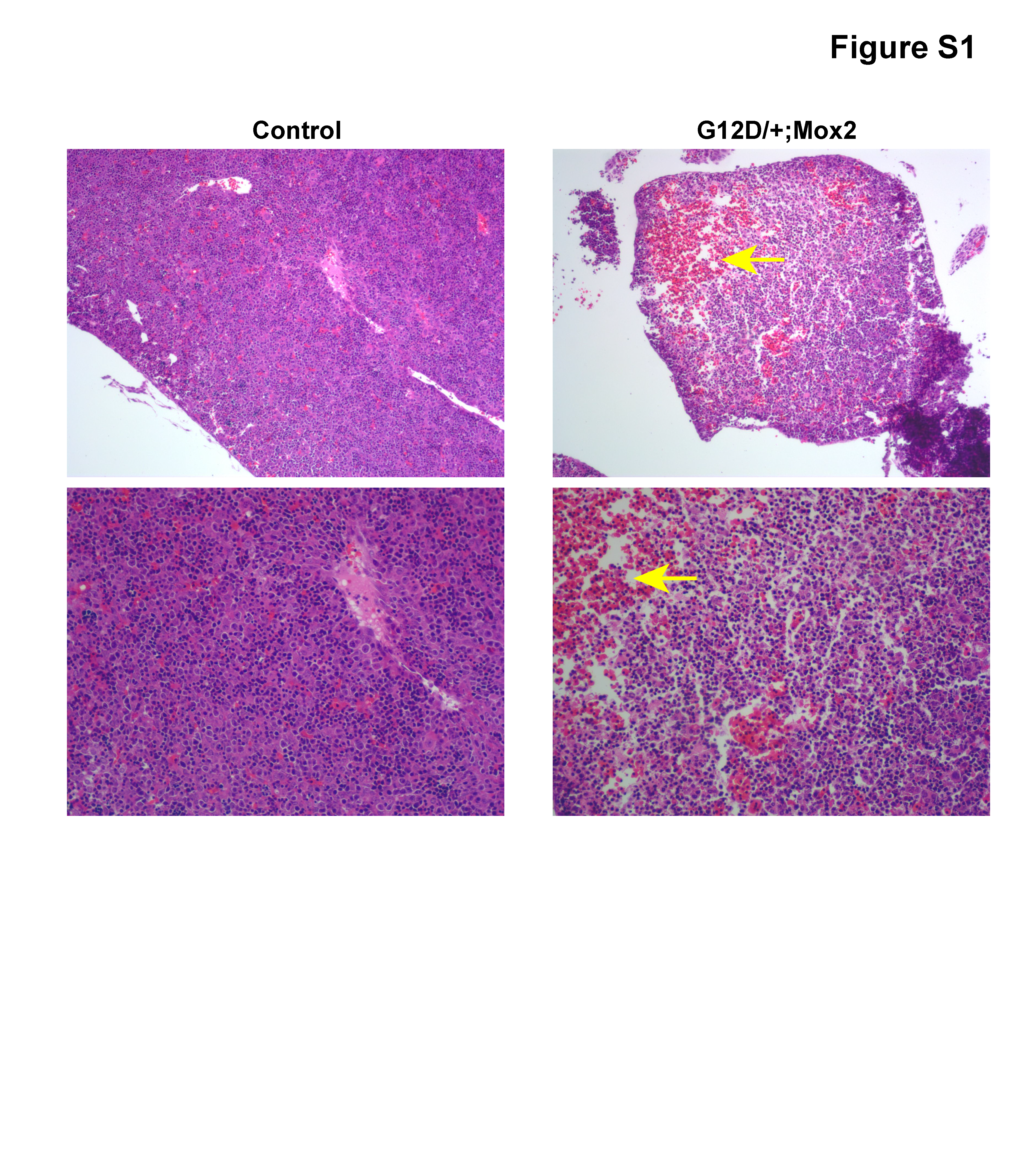

Supplement: Supplementary file 2 [file Image_1.TIFF]

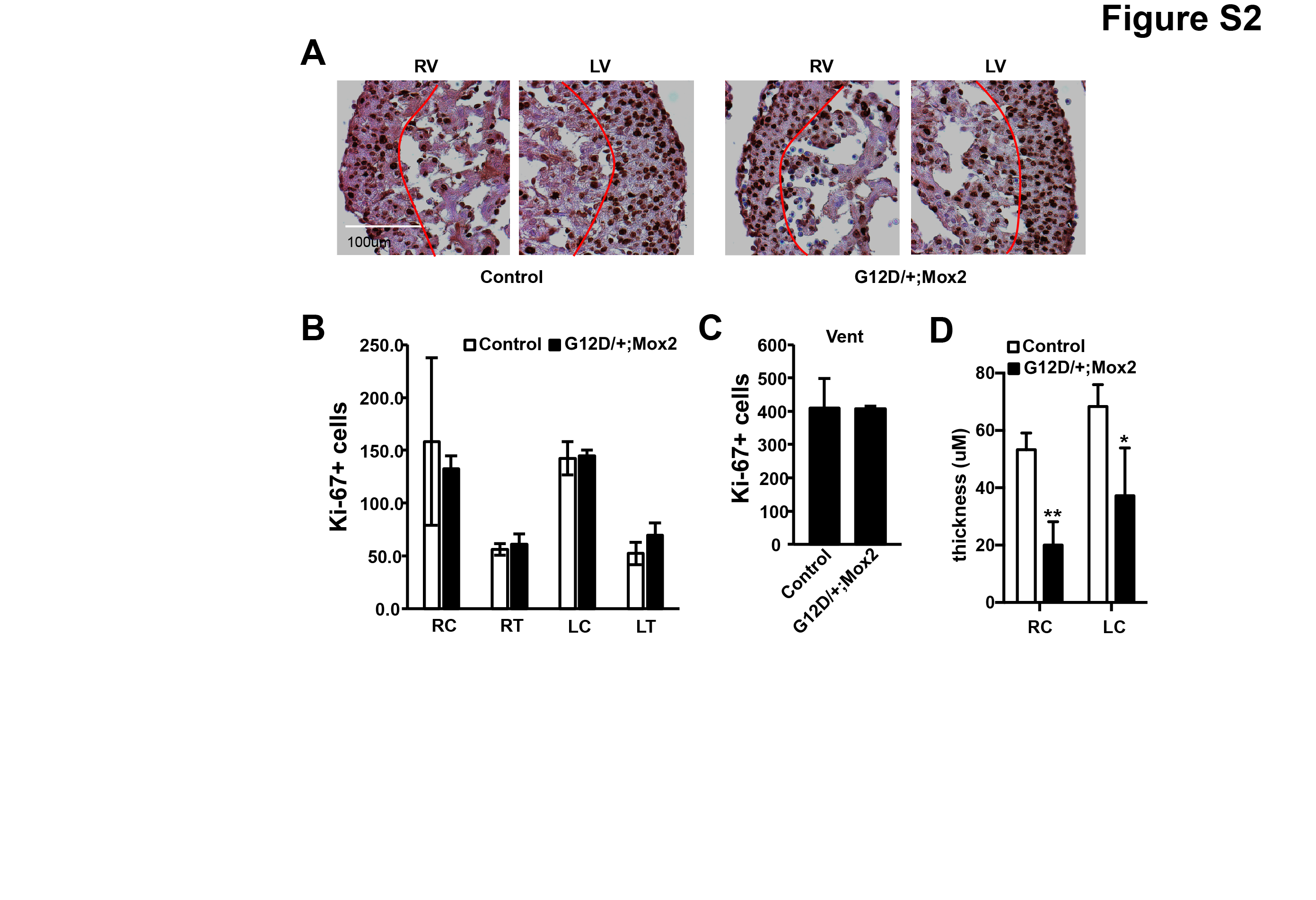

Supplement: Supplementary file 3 [file Image_2.TIF]

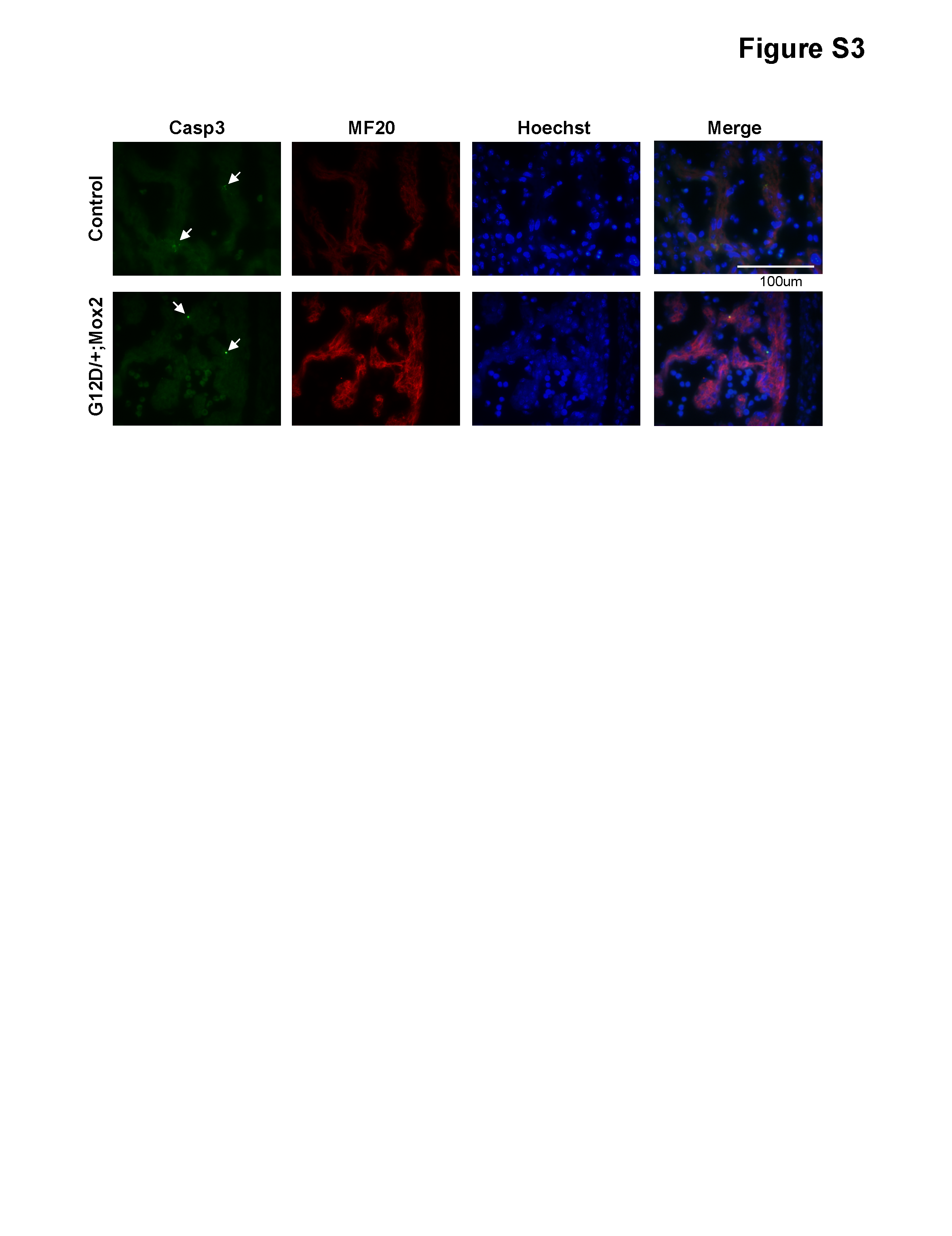

Supplement: Supplementary file 4 [file Image_3.TIFF]

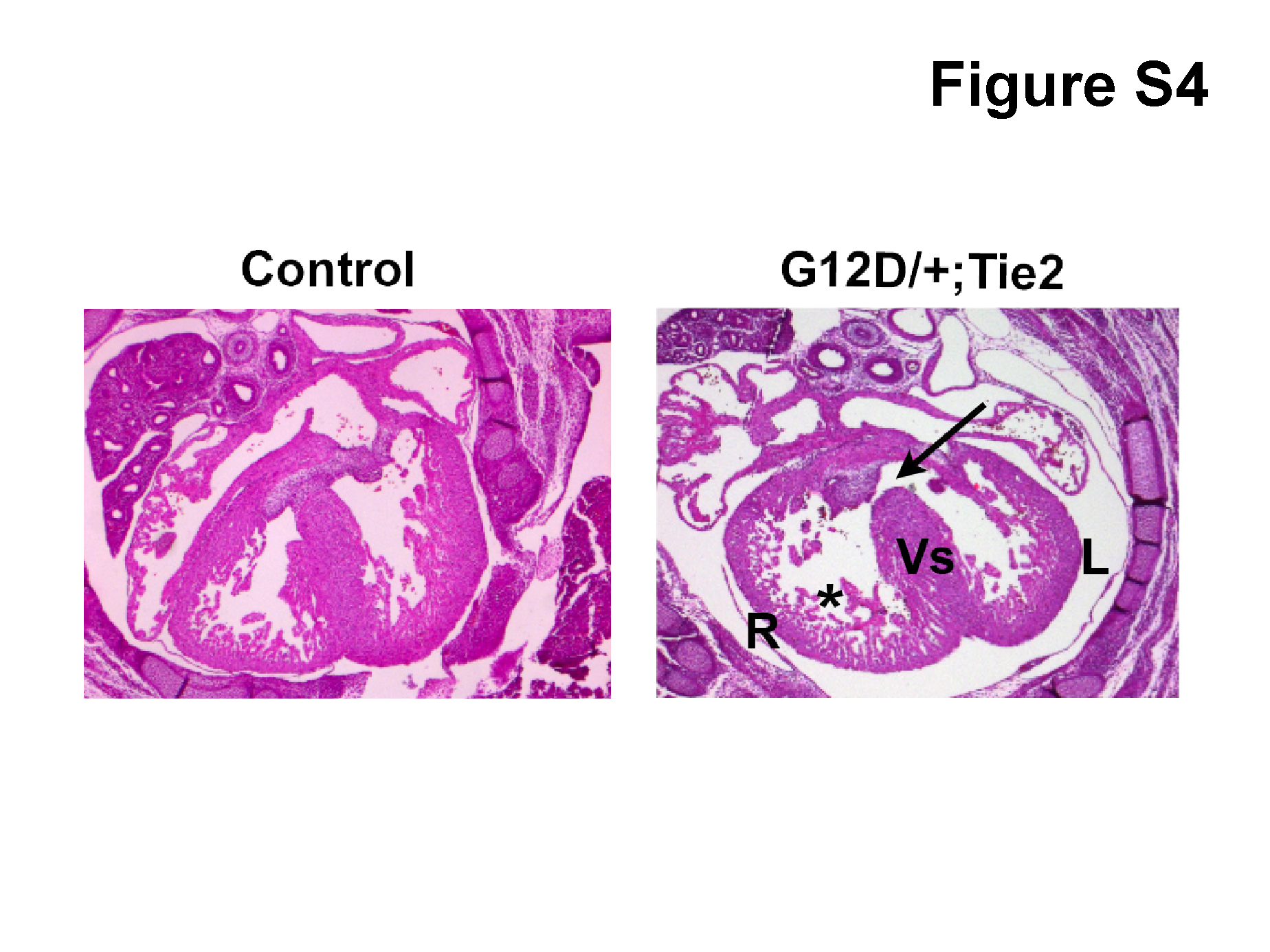

Supplement: Supplementary file 5 [file Image_4.TIFF]
